# Prognostic role of dynamic neutrophil-to-lymphocyte ratio in acute ischemic stroke after reperfusion therapy: A meta-analysis

**DOI:** 10.3389/fneur.2023.1118563

**Published:** 2023-02-16

**Authors:** Bing Wu, Fang Liu, Guiyan Sun, Shuang Wang

**Affiliations:** Department of Neurology, Army 78th Military Group Hospital, Mudanjiang, China

**Keywords:** acute ischemic stroke, neutrophil to lymphocyte ratio, endovascular therapy, reperfusion therapy, intravenous thrombolysis, prognostic

## Abstract

**Background:**

The prognostic role of the neutrophil-to-lymphocyte ratio (NLR), an inflammatory marker, in acute ischemic stroke (AIS) after reperfusion therapy remains controversial. Therefore, this meta-analysis sought to assess the correlation between the dynamic NLR and the clinical outcomes of patients with AIS after reperfusion therapy.

**Methods:**

PubMed, Web of Science, and Embase databases were searched to identify relevant literature from their inception to 27 October 2022. The clinical outcomes of interest included poor functional outcome (PFO) at 3 months, symptomatic intracerebral hemorrhage (sICH), and 3-month mortality. The NLR on admission (pre-treatment) and post-treatment was collected. The PFO was defined as a modified Rankin scale (mRS) of >2.

**Results:**

A total of 17,232 patients in 52 studies were included in the meta-analysis. The admission NLR was higher in the 3-month PFO (standardized mean difference [SMD] = 0.46, 95% confidence interval [CI] = 0.35–0.57), sICH (SMD = 0.57, 95% CI = 0.30–0.85), and mortality at 3 months (SMD = 0.60, 95% CI = 0.34–0.87). An elevated admission NLR was associated with an increased risk of 3-month PFO (odds ratio [OR] = 1.13, 95% CI = 1.09–1.17), sICH (OR = 1.11, 95% CI = 1.06–1.16), and mortality at 3 months (OR = 1.13, 95% CI = 1.07–1.20). The post-treatment NLR was significantly higher in the 3-month PFO (SMD = 0.80, 95% CI = 0.62–0.99), sICH (SMD = 1.54, 95% CI = 0.97–2.10), and mortality at 3 months (SMD = 1.00, 95% CI = 0.31–1.69). An elevated post-treatment NLR was significantly associated with an increased risk of 3-month PFO (OR = 1.25, 95% CI = 1.16–1.35), sICH (OR = 1.14, 95% CI = 1.01–1.29), and mortality at 3 months (OR = 1.28, 95% CI = 1.09–1.50).

**Conclusion:**

The admission and post-treatment NLR can be used as cost-effective and easily available biomarkers to predict the 3-month PFO, sICH, and mortality at 3 months in patients with AIS treated with reperfusion therapy. The post-treatment NLR provides better predictive power than the admission NLR.

**Systematic review registration:**

https://www.crd.york.ac.uk/PROSPERO/, identifier: CRD42022366394.

## Introduction

Acute ischemic stroke (AIS) is one of the major causes of disability and death in the world (1). Reperfusion therapy after AIS, including intravenous thrombolysis (IVT) and endovascular treatment (EVT), has been shown to effectively improve neurologic outcomes in eligible patients with AIS (2, 3). Nevertheless, approximately 50% of patients remain disabled or die 3 months after treatment (4). Age, infarct volume, hemorrhagic transformation, and baseline National Institutes of Health Stroke Scale (NIHSS) score are known major risk factors and predictors of adverse prognosis in patients with AIS (5, 6). However, the aforementioned risk factors as predictors of patient prognosis remain insufficient.

Recent studies have shown that inflammation plays an important role in stroke-induced injury, and elevated levels of inflammatory markers are associated with poor clinical outcomes (7–9). During the early stages of stroke, neutrophils accumulate in the ischemic area and release inflammatory mediators, leading to disruption of the blood–brain barrier (BBB), increased infarct volume, hemorrhagic transformation, and poor neurologic outcomes (8, 10). By contrast, lymphocytes as the brain's primary regulator may contribute to the repair of inflammatory damage as well as brain functional recovery (11). An increased infarct size and a worsening neurologic prognosis may be associated with the suppression of lymphocytes (8, 12). The neutrophil-to-lymphocyte ratio (NLR), a readily available serum biomarker for assessing the balance between neutrophils and lymphocytes, has been used to measure systemic inflammation (13, 14).

Previous retrospective cohort studies have shown that higher levels of admission or post-treatment NLR are associated with hemorrhagic transformation (HT) (15, 16), symptomatic intracerebral hemorrhage (sICH) (17–20), 3-month poor functional outcome (PFO) (17, 19, 21, 22), and mortality at 3 months (18, 19, 22) in patients with AIS treated with reperfusion therapy. Nevertheless, there is still no full understanding of the association between the dynamic NLR and clinical outcomes in patients with AIS treated with reperfusion therapy, owing to methodological limitations. In addition, several recent meta-analyses have also confirmed a link between the NLR and clinical outcomes in patients with AIS receiving reperfusion therapy (23–25). However, most of these reviews have certain limitations, such as the small number of included studies, inconsistent outcomes, different effect sizes, and different time points of NLR. Thus, we performed a meta-analysis to evaluate the association between the dynamic NLR and clinical outcomes in patients with AIS receiving reperfusion therapy.

## Methods

This systematic review and meta-analysis followed the Preferred Reporting Items for Systematic Reviews and Meta-Analyses (PRISMA) guidelines. The study protocol was registered with PROSPERO (number CRD42022366394).

### Search strategy and study selection

PubMed, Embase, and Web of Science were searched from their inception to 27 October 2022. The language of publication was limited to English. The following search terms were used: (“stroke”[All Fields] OR “brain infarction”[All Fields] OR “cerebral infarction”[All Fields] OR “ischemic stroke”[All Fields] OR “acute ischemic stroke”[All Fields]) AND (“neutrophil lymphocyte ratio”[All Fields] OR “neutrophil-to-lymphocyte ratio”[All Fields] OR “NLR”[All Fields]) AND (“tissue plasminogen activator”[All Fields] OR “recombinant tissue plasminogen activator”[All Fields] OR “tPA”[All Fields] OR “t-PA”[All Fields] OR “rtPA”[All Fields] OR “rt-PA”[All Fields] OR “alteplase”[All Fields] OR “thrombolysis”[All Fields] OR “endovascular thrombectomy”[All Fields] OR “mechanical thrombectomy”[All Fields] OR “thrombectomy”[All Fields] OR “endovascular treatment”[All Fields] OR “endovascular therapy”[All Fields] OR “reperfusion therapy”[All Fields]). Two investigators (BW and FL) independently assessed the titles and abstracts of the records and excluded articles that did not meet the eligibility criteria. Subsequently, the reviewers assessed the full-text articles. In addition, we manually reviewed the reference lists and recent reviews to identify potentially relevant studies.

Eligible studies met the following inclusion criteria: (1) patients with AIS who received reperfusion therapy with IVT or EVT after the symptom onset; (2) assessed the relationship between NLR and 3-month PFO, sICH, or mortality at 3 months after reperfusion therapy; (3) PFO was defined as the modified Rankin scale (mRS) >2; (4) blood samples were collected on admission (pre-treatment) or post-treatment; (5) studies with sufficient data for calculating standardized mean difference (SMD) and/or odds ratio (OR) with corresponding 95% confidence interval (CI); and (6) full text was available. We excluded studies if they met any one of the following criteria: (1) studies focused on a specific population with inflammatory disorders, infectious diseases, or any other major illness (such as cancer); (2) articles in the format of abstract, letter, meta-analysis, review, comment, case report, or editorial; (3) cell or animal research; (4) designated outcome was unreported; and (5) duplicate publications. For duplicate reports, the study with the largest sample size was selected.

### Data extraction and quality assessment

Two investigators (BW and FL) independently extracted the relevant data. The following information was extracted from each eligible study: first author, year of publication, country, study duration, study design, sample size, number of males, age, admission NIHSS score, treatment method, number and percentage of bridging therapy, blood collection time, study outcome, NLR cutoff, sICH definition, NOS scores, and whether the infection was excluded. If studies reported multiple post-treatment collection time points, we selected the time point closest to 24 h.

The methodological quality of the included studies was assessed using the Newcastle–Ottawa Scale (NOS) (26). The score ranges from 0 to 9, and studies with scores >7 are considered to be of high quality. Any disagreements regarding data extraction and quality assessment were resolved through consensus discussion.

### Statistical analysis

The pooled OR and SMD with 95% CI were used to analyze the association between NLR and 3-month PFO, sICH, or mortality at 3 months after reperfusion therapy. If the study provided only the median, range, or interquartile range (IQR), the mean and standard deviation (SD) values were estimated using the methods described by Luo et al. (27) and Wan et al. (28). When both adjusted and unadjusted OR were available, adjusted OR was used. The unadjusted OR was calculated when only count data were provided. The I^2^ statistic and the chi-square test were used to assess statistical heterogeneity among studies (29). A *p*-value of the chi-square test < 0.10 or I^2^ ≥ 50% was regarded as significant statistical heterogeneity. Considering the heterogeneity among the included studies, the random effects model (DerSimonian–Laird) was used to calculate the pooled effect sizes and the corresponding 95% CI. If sufficient studies were included (≥10) (30), subgroup analyses were conducted to explore the potential sources of heterogeneity according to the treatment method (EVT vs. IVT), study region (Asian vs. non-Asian), study design (prospective vs. retrospective), age (≥65 vs. < 65 years), sample size (≥200 vs. < 200), admission NIHSS score (≥15 vs. < 15), bridging therapy (≥40 vs. < 40%), OR (adjusted vs. unadjusted), NOS score (≥8 vs. < 8), cutoff for NLR (yes vs. no), and infection excluded (yes vs. no vs. not reported). Sensitivity analyses were conducted to test the robustness of the results by excluding each study sequentially (*n* ≥ 10). Egger's test and funnel plots were used to assess publication bias (*n* ≥ 10) (31). An analysis of trim and fill was performed to further evaluate the potential existence of publication bias. An SMD of 0.2 was interpreted as reflecting small effects, 0.5 as reflecting medium effects, and 0.8 as reflecting large effects according to Cohen's rule of thumb (32). All tests were two-sided, and *p* < 0.05 were considered statistically significant. All statistical analyses were performed using Stata 17 software (Stata Corporation LP, College Station, TX, USA).

## Results

### Study selection and characteristics

**Figure 1:**
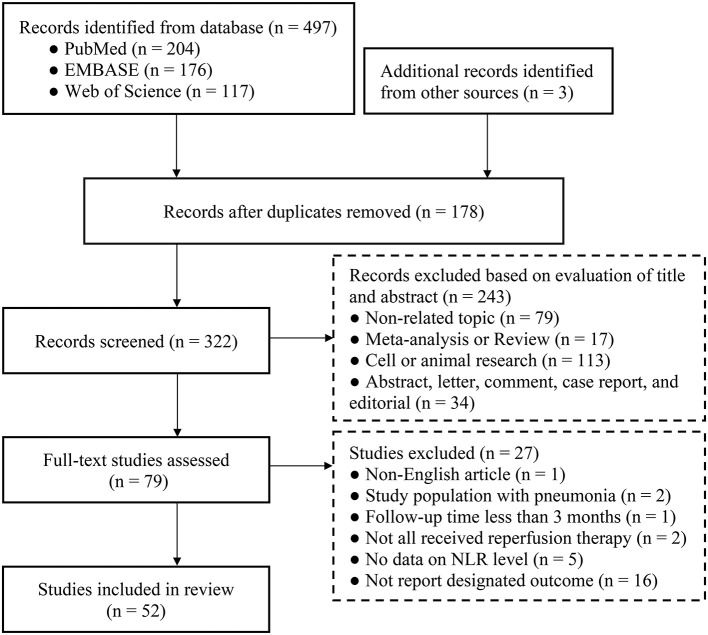
Flow diagram of the study search and selection process.

A primary literature search identified 497 potentially relevant articles. Three additional records were obtained from other sources. After removing 178 duplicate publications, the titles and abstracts of 322 studies were reviewed. We excluded 243 studies based on title and abstract reviews. Next, the full text of the remaining 79 articles was reviewed. Finally, 52 articles with 17,232 patients were included in our analysis (15–22, 33–76). The article selection process is illustrated in Figure 1.

The characteristics of the included studies and the quality assessment results are presented in Table 1. The selected studies were published between 2013 and 2022. The study design included single-center (*n* = 40), dual-center (*n* = 4), multi-center (*n* = 8), retrospective cohort (*n* = 48), and prospective cohort studies (*n* = 4). This meta-analysis included 23 studies treating AIS with IVT, 28 studies treating AIS with EVT, and two studies treating AIS with IVT/EVT (including IVT alone, EVT alone, and combination therapy of IVT and EVT). The sample sizes of the included studies ranged from 51 to 1,227 participants. The mean and/or median ages of the participants ranged from 58 to 75 years. The mean and/or median admission NIHSS score ranged from 4 to 27 points. Three IVT studies and 26 EVT studies reported percentages of bridging therapy ranging from 6.6% to 30.7% and 7.5% to 77.6%, respectively. Infection was excluded in 24 studies, not excluded in 10 studies, and was not reported in 18 studies. A total of 23 studies were considered high quality and reached a score of 8–9 points according to the NOS score.

**Table 1:** Basic characteristics of the studies included in the meta-analysis.

**ID**	**References**	**Country**	**Duration**	**Design**	**Sample size (male)**	**Age (year)**	**Admission NIHSS**	**Treatment**	**Bridging therapy, n (%)**	**Blood; collection; time**	**Study; outcome**	**NRL cut-off**	**sICH; definition**	**Infection excluded**	**NOS; scores**
1	Brooks et al. (21)	USA	2008–2011	R (S)	116 (NR)	67 (18–93)	17 (1–48)	EVT	27 (23.3)	Admission	(1) PFO; (2) Mortality	5.9	NR	No	7
2	Maestrini et al. (17)	France; Finland	NR	P (D)	846 (430)	71 (60–80)	10 (6–16)	IVT	56 (6.6)	Before IVT	(1) PFO; (2) sICH; (3) Mortality	4.8	ECASS II	No	9
3	Pagram et al. (33)	Australia	2009–2013	R (S)	141 (NR)	74.3 ± 10.7	10.1 ± 4.7	IVT	NP	Before IVT; after IVT	PFO	NR	NR	NR	8
4	Guo et al. (15)	China	2012–2015	R (D)	189 (123)	65.0 ± 10.6	12 (6–16)	IVT	58 (30.7)	Admission; after IVT	sICH	NR	ECASS II	Yes	9
5	Inanc et al. (34)	Turkey	2014–2015	R (S)	56 (35)	58.23 ± 11.94	16.09 ± 3.33	EVT	NR	Admission	(1) sICH; (2) Mortality	NR	NR	Yes	6
6	Goyal et al. (18)	USA	2012–2016	R (S)	293 (147)	62 ± 14	16 (13–19)	EVT	240 (70.0)	Admission	(1) PFO; (2) sICH; (3) Mortality	NR	SITS-MOST	NR	8
7	Wang et al. (35)	China	2014–2016	R (M)	199 (128)	64 (55–72)	16 (13–21)	EVT	85 (42.7)	Admission	PFO	NR	HBC	Yes	7
8	Duan et al. (36)	China	2014–2016	R (M)	616 (368)	66 (57–74)	16 (12–21)	EVT	216 (35.1)	Before EVT	(1) PFO; (2) sICH; (3) Mortality	7	HBC	Yes	8
9	Malhotra et al. (37)	USA	2011–2015	R (D)	657 (333)	64 ± 14	7 (4–13)	IVT	52 (7.9)	Before IVT	(1) PFO; (2) sICH; (3) Mortality	2.2	SITS-MOST	No	9
10	Shi et al. (38)	China	2009–2016	P (S)	372 (242)	64	10.9 ± 6.8	IVT	NP	Before IVT; after IVT	(1) PFO; (2) Mortality	NR	ECASS II	Yes	8
11	Semerano et al. (39)	Spain	2008–2017	R (M)	433 (266)	71 (61–80)	17 (11–21)	EVT	213 (49.0)	Before EVT; after EVT	(1) PFO; (2) sICH; (3) Mortality	NR	ECASS II	No	7
12	Pektezel et al. (40)	Turkey	2009–2018	R (S)	142 (62)	69 ± 13	13.9 ± 5.5	IVT	NP	Before IVT; after IVT	(1) PFO; (2) sICH	NR	ECASS II	No	6
13	Li et al. (41)	China	2017–2019	R (S)	156 (107)	64.43 ± 12.60	13 (11–17)	EVT	66 (42.31)	Admission	sICH	NR	ECASS III	NR	7
14	Ying et al. (42)	China	2016–2018	R (S)	208 (129)	67.3 ± 12.4	NR	IVT	NP	Admission; after IVT	(1) PFO; (2) sICH	NR	ECASS II	Yes	8
15	Meng et al. (43)	China	2015–2019	P (S)	302 (171)	69 ± 11	14.4 ± 4.6	EVT	115 (38.1)	Admission	(1) PFO; (2) Mortality	6.45	HBC	No	8
16	Lv et al. (44)	China	2016–2019	R (S)	564 (409)	63.0 (54.0–70.8)	8 (5–12)	IVT	NP	Before IVT	PFO	NR	NR	NR	7
17	Oh et al. (45)	Korea	2014–2019	R (S)	411 (222)	69.2 ± 13.4	10.4 ± 6.6	EVT	152 (37.0)	Before EVT	(1) PFO; (2) sICH; (3) Mortality	5.1	ECASS III	Yes	8
18	Cheng et al. (46)	China	2016–2019	R (S)	381 (253)	68 (59–76)	7.0 (3.5–11.0)	IVT	NP	after IVT	PFO	NR	NR	Yes	7
19	Liu et al. (47)	China	2016–2019	R (S)	192 (138)	60.8 ± 11.7	5.0 (3.0–6.8)	IVT	NP	Before IVT	PFO	3.9	NR	Yes	7
20	Switońska et al. (48)	Poland	2017–2018	R (S)	51 (22)	67 (55–78)	11 (6–16)	IVT/EVT	17 (33.0)	Admission	sICH	NR	ECASS II	Yes	6
21	Ozgen et al. (49)	Turkey	2017–2018	P (S)	150 (83)	NR	NR	EVT	NP	Admission	(1) PFO; (2) Mortality	NR	NR	Yes	7
22	Pan et al. (50)	China	2016–2018	R (S)	151 (97)	68 (59–74)	9 (6–14)	IVT	NP	Admission; after IVT	PFO	NR	NR	Yes	7
23	Lux et al. (51)	UK	2016–2017	R (S)	121 (58)	66.4 ± 16.7	19 (1–28)	EVT	94 (77.6)	Admission; after EVT	PFO	NR	ECASS I	Yes	8
24	Aly et al. (19)	USA	2015–2019	R (S)	142 (69)	70 ± 16	17 (12–21)	EVT	70 (49.0)	Admission; after EVT	(1) PFO; (2) sICH; (3) Mortality	NR	ECASS III	Yes	7
25	Hu et al. (52)	China	2014–2019	R (S)	183 (123)	64.9 ± 10.5	4 (3–7)	IVT	NP	after IVT	PFO	NR	NR	Yes	8
26	Topcuoglu et al. (53)	Turkey	2011–2019	R (S)	165 (96)	70 ± 14	13 ± 5.6	IVT	NP	Before IVT; after IVT	(1) PFO; (2) sICH	NR	ECASS I	Yes	6
27	Majid et al. (54)	Pakistan	2015–2019	R (S)	98 (60)	58.0 ± 6.4	15.9 ± 7.7	IVT	NP	Admission	PFO	2.39	NR	Yes	6
28	Chen et al. (55)	China	2015–2019	R (S)	257 (186)	63.2 ± 12.6	NR	EVT	115 (44.7)	Admission	(1) PFO; (2) sICH	NR	ECASS II	Yes	8
29	Ören et al. (20)	Turkey	2016–2018	R (S)	133 (81)	66.56 ± 12.47	13 (10–17)	IVT	NR	Admission	sICH	NR	ECASS III	Yes	8
30	Guo et al. (56)	China	2014–2020	R (M)	1200 (756)	66.9 ± 12.5	NR	IVT	NP	Admission	sICH	NR	ECASS II	Yes	9
31	Yu et al. (57)	China	2018–2020	R (S)	102 (54)	66.9 ± 13.89	13.5 (9.75–17.00)	EVT	33 (32.3)	Admission	sICH	NR	HBC	NR	7
32	Weng et al. (58)	China	2016–2019	R (S)	291 (104)	67 (59–79)	8 (5–13)	IVT	NP	Admission; after IVT	Mortality	NR	NR	NR	7
33	Ferro et al. (59)	Portugal	2017–2019	R (S)	325 (161)	75 (66–83)	14 (8–19)	IVT/EVT	87 (26.7)	After IVT (EVT)	PFO	NR	NR	No	8
34	Yi et al. (60)	Korea	2015–2020	R (S)	440 (260)	70.2 ± 12.9	NR	EVT	159 (36.1)	Admission	PFO	3.7	ECASS III	Yes	7
35	Chen et al. (61)	China	2016–2019	R (S)	280 (179)	69 (59–77)	NR	IVT	NP	Admission	(1) PFO; (2) Mortality	NR	NR	NR	7
36	Xie et al. (62)	China	2014–2020	R (S)	462 (318)	63.3 ± 12.5	NR	IVT	NP	Before IVT	sICH	NR	ECASS II	NR	8
37	Yang et al. (63)	China	2016–2020	R (D)	623 (403)	67.36 ± 12.84	6 (4–10)	IVT	NP	Admission; after IVT	(1) PFO; (2) Mortality	NR	NR	Yes	6
38	Shi et al. (64)	China	2015–2017	R (S)	127 (65)	70.95 ± 12.24	20 (16–25)	EVT	53 (41.7)	Admission; after EVT	PFO	NR	HBC	NR	7
39	Gao et al. (65)	China	2016–2019	R (S)	283 (180)	NR	NR	IVT	NP	Before IVT	PFO	NR	NR	NR	7
40	Li et al. (66)	China	2018–2020	R (S)	286 (167)	70 (63–77)	18 (12–30)	EVT	41 (14.3)	Before EVT	PFO	NR	ECASS II	NR	8
41	Lee et al. (67)	Korea	2015–2011	R (M)	282 (162)	69.5 ± 13.4	NR	EVT	148 (52.5)	Before EVT	PFO	NR	NR	NR	6
42	Sun et al. (68)	China	2017–2019	R (M)	147 (75)	67 (59–75)	16 (11–19)	EVT	302 (29.3)	Admission	PFO	4.1	HBC	NR	8
43	Zou et al. (69)	China	2017–2020	R (S)	160 (101)	70 (64–76)	15 (11–20)	EVT	12 (7.5)	After EVT	PFO	9.75	HBC	Yes	6
44	Weyland et al. (70)	Germany	2014–2019	R (S)	549 (273)	74.3 ± 12.6	NR	EVT	307 (55.9)	Admission	PFO	NR	NR	NR	8
45	Liao et al. (22)	China	2014–2019	R (M)	586 (443)	64 (56–73)	27 (17–33)	EVT	109 (18.6)	Admission	(1) PFO; (2) Mortality	NR	HBC	No	8
46^*^	Chen et al. (71)	China	2018–2020	R (S)	576 (379); 351 (249)	68 (59–76); 69 (60–76)	5 (3–10); 14 (11–19)	IVT; EVT	NP; 88 (25.1)	Before IVT/EVT; after IVT/EVT	(1) PFO; (2) sICH; (3) Mortality	NR	ECASS II	No	8
47	Shen et al. (72)	China	2019–2020	R (S)	369 (250)	66 (57–74)	15 (12–19)	EVT	80 (21.7)	Admission	sICH	5.48	HBC	NR	7
48	Sun et al. (73)	China	2017–2019	R (M)	1227 (776)	66 (56–73)	16 (12–20)	EVT	357 (29.1)	Admission	sICH	NR	ECASS II	NR	7
49	Sadeghi et al. (74)	Hungary	2016–2018	R (S)	285 (159)	66 ± 12.9	6.0 (5.0–9.1)	IVT	NP	Before IVT; after IVT	sICH	NR	ECASS II	NR	6
50	Kim et al. (75)	Korea	2013–2019	R (S)	128 (67)	68.9 ± 13.2	17 (13–21)	EVT	50 (39.1)	Before EVT; after EVT	sICH	NR	SITS-MOST	Yes	7
51	Li et al. (16)	China	2015–2021	R (S)	258 (156)	70 s(61–79)	NR	EVT	88 (34.1)	Before EVT; after EVT	PFO	NR	ECASS III	NR	8
52	Feng et al. (76)	China	2019–2021	R (S)	170 (115)	66.0 (58.8–74.3)	15 (12–19)	EVT	62 (36.5)	Admission; after EVT	PFO	NR	NR	No	7

NOS, Newcastle–Ottawa Scale; NIHSS, National Institute of Health Stroke Scale; sICH, symptomatic intracranial hemorrhage; PFO, poor functional outcome; ECASS, European Cooperative Acute Stroke Studies; SITS-MOST, Safe Implementation of Thrombolysis in Stroke-Monitoring Study; HBC, Heidelberg Bleeding Classification; R, retrospective cohort; P, prospective cohort; S, single-center; B, dual-center; M, multi-center; NP, bridging therapy not performed; NR, not reported; IVT, intravenous thrombolysis; EVT, endovascular treatment; NRL, neutrophil-to-lymphocyte ratio.

^*^This study reported separately on the IVT and EVT groups.

### Relationship between admission NLR and 3-month PFO

**Figure 2:**
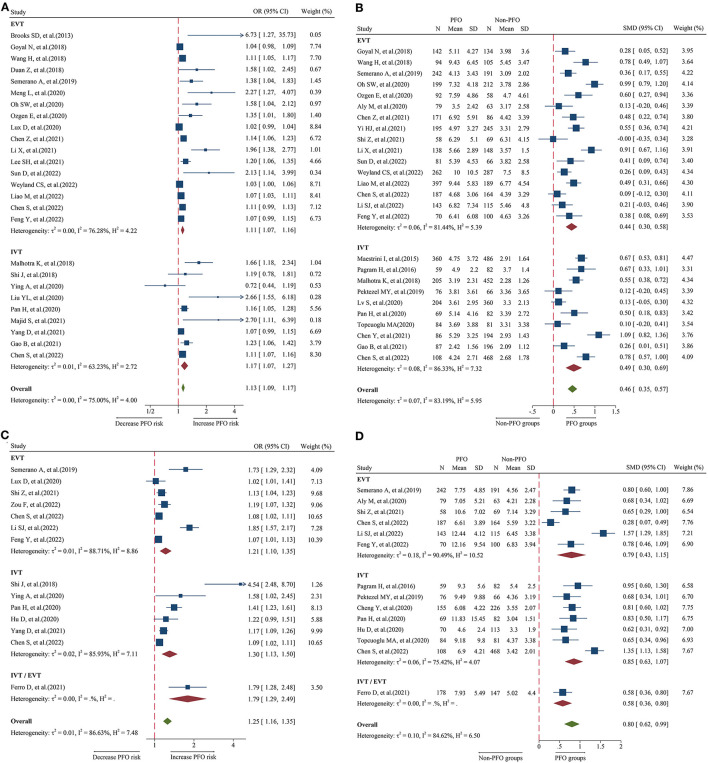
Forest plot showing the association of NLR and 3-month PFO; **(A)** forest plot of admission NLR based on OR; **(B)** forest plot of admission NLR based on SMD; **(C)** forest plot of post-treatment NLR based on OR; and **(D)** forest plot of post-treatment NLR based on SMD.

A total of 26 studies including 8,474 patients were used for the pooled OR analysis. Higher admission NLR levels were associated with an increased risk of 3-month PFO (OR = 1.13, 95% CI = 1.09–1.17, I^2^ = 75.0%) (Figure 2A). The summary effect sizes in the IVT (OR = 1.17, 95% CI = 1.07–1.27) and EVT (OR = 1.11, 95% CI = 1.07–1.16) groups remained significant. Subgroup analyses indicated no evidence of heterogeneity among groups (Supplementary Table 1). A possible publication bias was detected by the visual inspection of the funnel plot (**Figure 5A**) and Egger's test (*P* < 0.001). The result remained significant for the association between admission NLR levels and PFO (OR = 1.10, 95% CI = 1.04–1.14) after the trim-and-fill analysis imputed 10 theoretical missing studies. Moreover, we performed sensitivity analyses, and the cumulative results remained steady after sequentially excluding each study (**Figure 6A**).

A total of 26 studies including 7,743 patients were used for the pooled SMD analysis. Patients with PFO had higher levels of admission NLR than patients without PFO (SMD = 0.46, 95% CI = 0.35–0.57, I^2^ = 83.1%) (Figure 2B). Similar results were observed in IVT (SMD = 0.49, 95% CI = 0.30–0.69) and EVT patients (SMD = 0.44, 95% CI = 0.30–0.58). We conducted several subgroup analyses and found no evidence of heterogeneity (Supplementary Table 2). The visual inspection of the funnel plot (**Figure 5B**) and Egger's test (*P* = 0.48) showed no evidence of publication bias. No significant change was observed in the pooled SMD after excluding each study (**Figure 6B**).

### Relationship between post-treatment NLR and 3-month PFO

A total of 14 studies including 3,686 patients were used for the pooled OR analysis. Higher post-treatment NLR levels were associated with an increased risk of PFO (OR = 1.25, 95% CI = 1.16–1.35, I^2^ = 86.6%) (Figure 2C). The relationship remained significant in the IVT (OR = 1.30, 95% CI = 1.13–1.50) and EVT groups (OR = 1.21, 95% CI = 1.10–1.35). A significant result was also obtained in one study that included IVT/EVT (OR = 1.79, 95% CI = 1.29–2.49). No source of heterogeneity was found in the subgroup analyses (Supplementary Table 3). According to the funnel plot (**Figure 5C**) and Egger's test (*P* < 0.001), there was a potential publication bias. After trimming and filling in four theoretically missing studies, the relationship between post-treatment NLR levels and 3-month PFO remained significant (OR = 1.15, 95% CI = 1.06–1.25). In the sensitivity analyses, the pooled OR was not significantly affected by excluding individual studies (**Figure 6C**).

A total of 14 studies including 3,380 patients were used for pooled SMD analysis. Post-treatment NLR levels were higher in patients with PFO than in those without PFO (SMD = 0.80, 95% CI = 0.62–0.99, I^2^ = 84.6%) (Figure 2D). Similar results were also achieved in the IVT (SMD = 0.85, 95% CI = 0.63–1.07) and EVT groups (SMD = 0.79, 95% CI = 0.43–1.15). IVT/EVT was included in only one study (SMD = 0.58, 95% CI = 0.36–0.80), and the result was also significant. According to the results of subgroup analyses, no source of heterogeneity was found (Supplementary Table 4). No substantial publication bias was found, according to the funnel plot (**Figure 5D**) and Egger's test (*P* = 0.96). In the sensitivity analyses, the results implied that no studies had a significant effect on the pooled SMD (**Figure 6D**).

### Relationship between admission NLR and sICH

**Figure 3:**
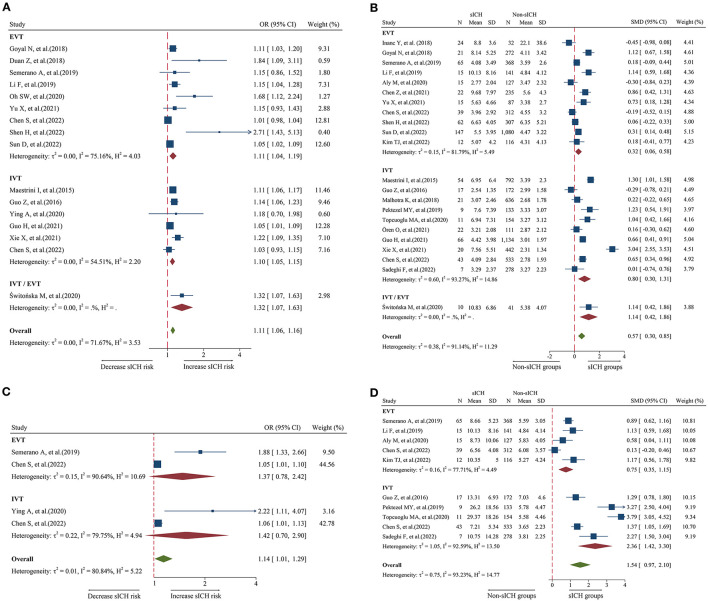
Forest plot showing the association of NLR and sICH; **(A)** forest plot of admission NLR based on OR; **(B)** Forest plot of admission NLR based on SMD; **(C)** Forest plot of post-treatment NLR based on OR; and **(D)** forest plot of post-treatment NLR based on SMD.

A total of 16 studies including 6,977 patients were used for the pooled OR analysis. Higher admission NLR levels were associated with an increased risk of sICH (OR = 1.11, 95% CI = 1.06–1.16, I^2^ = 71.6%) (Figure 3A). Compared with the main analysis, the results of the IVT (OR = 1.10, 95% CI = 1.05–1.15) and EVT (OR = 1.11, 95% CI = 1.04–1.19) groups were generally consistent. Only one study included IVT/EVT (OR = 1.32, 95% CI = 1.07–1.63), and the result was also significant. No source of heterogeneity was found in subgroup analyses (Supplementary Table 5). Potential publication bias was detected by a visual inspection of the funnel plot (**Figure 5E**) and Egger's test (*P* < 0.001). There was still a significant relationship between admission NLR and sICH after trimming and filling in seven theoretically missing studies (OR = 1.08, 95% CI = 1.03–1.12). According to the results of the sensitivity analyses, no study had a significant effect on the pooled OR (**Figure 6E**).

A total of 22 studies including 8,055 patients were used for the pooled SMD analysis. The result suggested a difference between sICH and non-sICH groups (SMD = 0.57, 95% CI = 0.30–0.85, I^2^ = 91.1%) (Figure 3B). The results of the IVT (SMD = 0.80, 95% CI = 0.30–1.31) and EVT (SMD = 0.32, 95% CI = 0.06–0.58) groups were generally in accordance with those of the main analysis. Only one study included IVT/EVT, and the result was significant (SMD = 1.14, 95% CI = 0.42–1.86). We performed several subgroup analyses and found no evidence of heterogeneity (Supplementary Table 6). Egger's test (*P* = 0.53) and a visual examination of the funnel plot (**Figure 5F**) indicated no evidence of publication bias. According to the results of the sensitivity analyses, none of the studies greatly impacted the pooled SMD (**Figure 6F**).

### Relationship between post-treatment NLR and sICH

A total of four studies including 1,568 patients were used for the pooled OR analysis. There was an association between higher post-treatment NLR levels and a higher risk of sICH (OR = 1.14, 95% CI = 1.01–1.29, I^2^ = 80.8%) (Figure 3C). However, these findings were not replicated in the IVT (OR = 1.42, 95% CI = 0.70–2.90) and EVT groups (OR = 1.37, 95% CI = 0.78–2.42). Publication bias, sensitivity, and subgroup analyses were not performed because of the small number of studies.

A total of 10 studies including 2,402 patients were used for the pooled SMD analysis. Post-treatment NLR was higher in patients with sICH than in those without sICH (SMD = 1.54, 95% CI = 0.97–2.10, I^2^ = 90.9%) (Figure 3D). Meanwhile, the results were consistent with the earlier findings in the IVT (SMD = 2.36, 95% CI = 1.42–3.30) and EVT groups (SMD = 0.75, 95% CI = 0.35–1.15). There was no evidence of heterogeneity among subgroup analyses (Supplementary Table 7). Egger's test (*P* = 0.005) and a visual examination of the funnel plot (**Figure 5G**) indicated evidence of publication bias. The meta-analysis results did not change after adjusting for publication bias using the trim-and-fill method. The results of the sensitivity analyses showed that none of the studies had a significant effect on the pooled SMD (**Figure 6G**).

### Relationship between admission NLR and 3-month mortality

**Figure 4:**
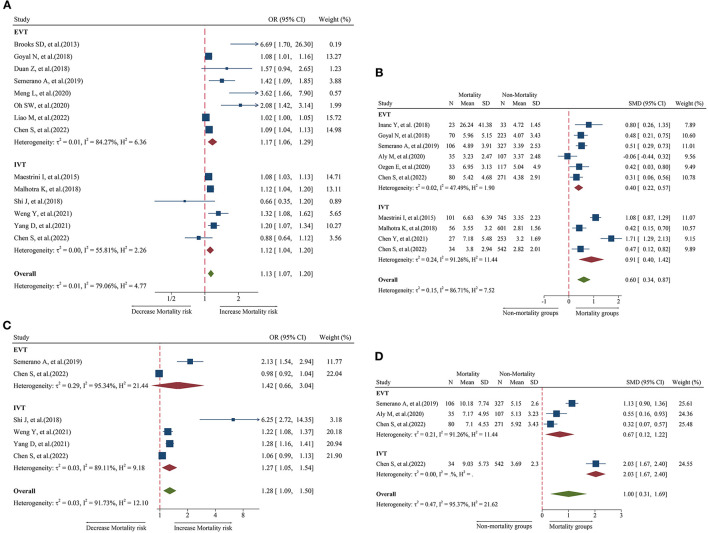
Forest plot showing the association of NLR and 3-month mortality; **(A)** forest plot of admission NLR based on OR; **(B)** forest plot of admission NLR based on SMD; **(C)** forest plot of post-treatment NLR based on OR; and **(D)** forest plot of post-treatment NLR based on SMD.

**Figure 5:**
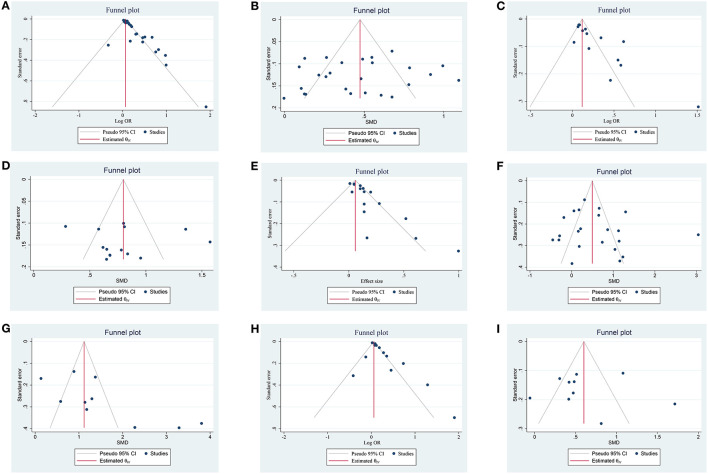
Funnel plot of the publication bias on the association of NLR and prognosis; **(A)** funnel plot of admission NLR and 3-month PFO based on OR; **(B)** funnel plot of admission NLR and 3-month PFO based on SMD; **(C)** funnel plot of post-treatment NLR and 3-month PFO based on OR; **(D)** funnel plot of post-treatment NLR and 3-month PFO based on SMD; **(E)** funnel plot of admission NLR and sICH based on OR, **(F)** funnel plot of admission NLR and sICH based on SMD; **(G)** funnel plot of post-treatment NLR and sICH based on SMD; **(H)** funnel plot of admission NLR and 3-month mortality based on OR; and **(I)** funnel plot of admission NLR and 3-month mortality based on SMD.

**Figure 6:**
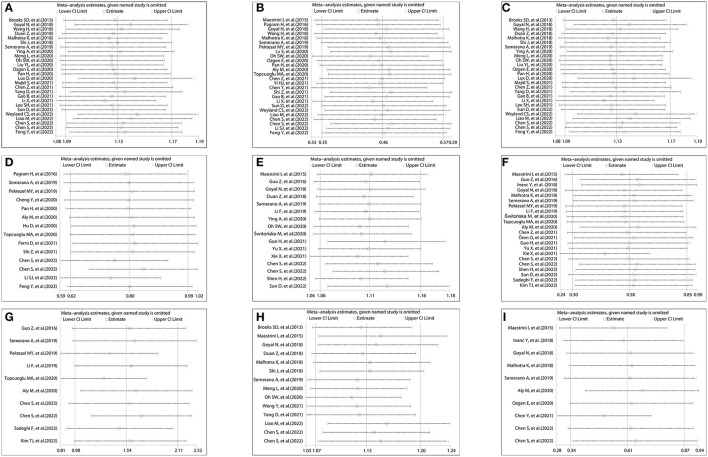
Sensitivity analysis on the relationship between NLR and prognosis; **(A)** sensitivity analysis of admission NLR and 3-month PFO based on OR; **(B)** sensitivity analysis of admission NLR and 3-month PFO based on SMD; **(C)** sensitivity analysis of post-treatment NLR and 3-month PFO based on OR; **(D)** sensitivity analysis of post-treatment NLR and 3-month PFO based on SMD; **(E)** sensitivity analysis of admission NLR and sICH based on OR; **(F)** sensitivity analysis of admission NLR and sICH based on SMD; **(G)** sensitivity analysis of post-treatment NLR and sICH based on SMD; **(H)** sensitivity analysis of admission NLR and 3-month mortality based on OR; and **(I)** sensitivity analysis of admission NLR and 3-month mortality based on SMD.

A total of 14 studies including 6,473 patients were used for the pooled OR analysis. Higher admission NLR levels were associated with an increased risk of mortality (OR = 1.13, 95% CI = 1.07–1.20, I^2^ = 79.0%) (Figure 4A). These findings were confirmed in the IVT (OR = 1.12, 95% CI = 1.04–1.20) and EVT groups (OR = 1.17, 95% CI = 1.06–1.29). Several subgroup analyses were conducted; however, no source of heterogeneity was found (Supplementary Table 8). There was possible publication bias according to the funnel plot (Figure 5H) and Egger's test (*P* < 0.001). After trimming and filling in three theoretically missing studies, admission NLR remained significantly related to mortality (OR = 1.11, 95% CI = 1.04–1.86). After excluding each study, the pooled OR did not change significantly (Figure 6H).

A total of 10 studies including 3,784 patients were used for the pooled SMD analysis. Admission NLR levels were higher in patients with mortality (SMD = 0.60, 95% CI = 0.34–0.87, I^2^ = 86.7%) than in those without mortality (Figure 4B). The results remained significant in IVT (SMD = 0.91, 95% CI = 0.40–1.42) and EVT groups (SMD = 0.40, 95% CI = 0.22–0.57). No cause of heterogeneity was found in the subgroup analyses (Supplementary Table 9). The visual inspection of the funnel plot (Figure 5I) and Egger's test (*P* = 0.56) showed no potential publication bias. In the sensitivity analyses, no studies significantly impacted the pooled SMD (Figure 6I).

### Relationship between post-treatment NLR and 3-month mortality

A total of six studies including 2,274 patients were used for the pooled OR analysis. Higher post-treatment NLR levels were associated with an increased risk of mortality (OR = 1.28, 95% CI = 1.09–1.50, I^2^ = 91.7%) (Figure 4C). This finding remained significant in the IVT group (OR = 1.27, 95% CI = 1.05–1.54). However, this relationship was not significant in the EVT group (OR = 1.42, 95% CI = 0.66–3.04). Publication bias, subgroup, and sensitivity analyses were not performed because our analysis included < 10 studies.

A total of four studies including 1,052 patients were used for the pooled SMD analysis. Post-treatment NLR levels were higher in patients with mortality (SMD = 1.00, 95% CI = 0.31–1.69, I^2^ = 95.4%) than in patients without mortality (Figure 4D). The results did not change in the IVT (SMD = 2.03, 95% CI = 1.67–2.40) and EVT (SMD = 0.67, 95% CI = 0.12–1.22) groups. The number of studies was too small to conduct subgroup analyses, sensitivity analyses, and publication bias.

## Discussion

This meta-analysis included 52 recent clinical studies with large sample sizes to investigate the association between the dynamic NLR and PFO at 3 months, sICH, and 3-month mortality in AIS after reperfusion therapy. We reported the results of both primary and secondary outcomes with effect sizes (OR or SMD) and 95% CIs. The results suggested that the higher levels of both admission and post-treatment NLR were associated with an increased risk of 3-month PFO, sICH, and mortality at 3 months, according to the pooled OR. Pooled SMD results showed that both admission and post-treatment NLR levels were higher in the PFO, sICH, and mortality groups than in the control group. Notably, post-treatment NLR showed better predictive capabilities for poor clinical outcomes of patients with AIS treated with reperfusion therapy than admission.

The role and mechanism of inflammation in the pathophysiology of AIS have been extensively studied (8). Ischemia and reperfusion damage can cause a marked inflammatory response, further increasing brain injury (8, 77). Recanalization treatment also leads to ischemia and reperfusion injury, which exacerbates acute brain injury and results in poor functional outcomes (77). After ischemic stroke, neutrophils migrate into cerebral ischemic regions within the first few hours after the onset of ischemia and activate the immune system (7, 9). Increased neutrophils destroy the BBB and increase cerebral edema and neurologic impairment by the activation of inflammatory mediators such as chemokines and cytokines, reactive oxygen species (ROS), and the release of adhesion molecules and proteolytic enzymes (7, 9). In summary, the pro-inflammatory activation of neutrophils increases infarct size, hemorrhagic transformation, and adverse neurologic outcomes (8, 9). Lymphocytes as the main leukocyte subpopulation may contribute to the repair of the ischemic brain tissue, in which regulatory T and B cells are important brain protective immunomodulators in ischemic stroke (11, 12). In addition, decreased lymphocyte counts may reflect a cortisol-induced stress response and a sign of reduced sympathetic tone, which may promote the secretion of pro-inflammatory cytokines, resulting in an increased risk of ischemia and reperfusion damage after ischemic stroke (75, 78). The NLR is considered to represent the balance between neutrophils and lymphocytes and has recently been reported as an easy computing, inexpensive, and stable comprehensive systemic inflammatory biomarker. Numerous studies have investigated the predictive and prognostic values of NLR in patients with AIS treated with IVT or EVT (15–22, 33–76). However, the conclusions of these studies are inconsistent. Meta-analysis provides a much more likely approach for reaching reasonably strong conclusions.

Several previous meta-analyses have reviewed the predictive value of NLR for PFO after reperfusion therapy in patients with AIS (23–25). A meta-analysis conducted by Bi et al. (24) included six studies and showed that an increased baseline NLR was associated with 3-month PFO. Another study by Sharma et al. (25), which included 13 studies, showed that a lower admission NLR was associated with good functional outcomes (mRS 0–2). Our meta-analysis, which included 26 studies for the pooled SMD analysis, further verified the earlier results. Furthermore, the current study is also in line with the meta-analysis by Sharma et al. (25), which demonstrated that post-treatment NLR was related to 3-month PFO. However, the pooled SMD could not be interpreted as a risk measure because the effect sizes were not adjusted for potential confounders. Hence, we included 26 and 14 studies separately for the pooled OR analysis to investigate the relationship between NRL and 3-month PFO at admission and post-treatment. The results showed that higher admission NLR and post-treatment NLR increased the risk of 3-month PFO. Due to the large SMD and the higher OR of post-treatment NLR, our findings demonstrated that post-treatment NLR levels displayed a stronger predictive power for 3-month PFO than admission. There are several possible mechanisms to explain the earlier findings. First, neutrophils infiltrate the ischemic brain between 30 min and a few hours after infarction, peaking between days 1 and 3, and then declining steadily thereafter (79). Second, 24–48 h after reperfusion, BBB disruption leads to increased intracranial pressure and vasogenic edema (79). Third, the regulatory lymphocyte level in the ischemic brain parenchyma is low during the 1st day after stroke (80).

This study demonstrated that admission and post-treatment NLR levels were higher in patients with sICH than in those without sICH after reperfusion therapy in the pooled SMD analysis. The overall results were consistent with the meta-analysis by Bi et al. (24) and Sharma et al. (25). However, Sharma et al. (25) did not detect a significant association between post-treatment NLR and sICH in subgroup analyses stratified by the treatment method, and Bi et al. (24) did not investigate the relationship between post-treatment NLR and sICH. Compared with the previous meta-analysis, we included 22 and 10 studies separately to limit selection and publication bias, which may have influenced the results. Furthermore, subgroup analyses based on the type of treatment also showed that higher levels of admission and post-treatment NLR were observed in patients with sICH than in those without sICH. Similarly, for the pooled OR analysis, we found that the higher levels of admission and post-treatment NLR increased the risk of sICH. However, interestingly, we did not find a relationship between post-treatment NLR and sICH in the subgroup analyses stratified by the treatment type. Fewer studies were included, and publication bias may explain this inconsistency. Further studies are needed to explore this causal relationship. Our findings suggested that post-treatment NLR levels had a stronger predictive power for sICH than admission due to its large SMD and higher OR. The possible underlying mechanism for these findings is that neutrophils enter the brain and release matrix metalloproteinase-9 (MMP-9), which may act on tight-junction proteins and then destroy the BBB from the lumen side of the blood vessels (77).

The present study also revealed that admission and post-treatment NLR levels were higher in patients with mortality at 3 months than in patients without mortality after reperfusion therapy in the pooled SMD analysis. These findings are consistent with those of the previous studies (25). However, in subgroup analyses stratified by the treatment type, Sharma et al. (25) did not find a statistically significant difference between admission NLR and 3-month mortality in patients with AIS treated with EVT ± IVT. In contrast, we included more studies than the aforementioned study, and the results indicated that a correlation exists between admission NLR and 3-month mortality in EVT patients. However, the pooled SMD only evaluated the differences in NLR levels between mortality and non-mortality. As a result, we also combined OR to assess whether higher NLR levels increased the risk of mortality. Our research showed that higher admission and post-treatment NLR levels were associated with an increased risk of mortality. However, higher post-treatment NLR was not associated with mortality in the EVT group. The possible reasons for this difference may include that only two studies met the inclusion criteria and had higher baseline NIHSS scores. Based on the large SMD and higher OR, we suggest that post-treatment NLR levels have greater predictive power for mortality than admission. These results may be related to the pathophysiological mechanisms described previously.

Several studies have shown that NLR as a dynamic variable is associated with HT (15, 16), sICH (15, 19, 40, 42, 71, 75), 3-month PFO (16, 19, 38, 40, 42, 51, 58, 63, 71, 75, 76), and death (19, 38, 58, 63, 71) in patients with AIS after IVT or EVT. These studies also showed that the post-treatment NLR has a more strong predictive ability for the poor prognosis of patients with AIS after reperfusion therapy than admission. The results of these previous studies are basically consistent with our findings.

Similar to other studies, this meta-analysis has some limitations. First, most of the included studies had a retrospective design, which made them vulnerable to selection bias and uncontrolled confounding factors. Therefore, future prospective cohort studies with adjustments for potential confounders are required to further explore the possible impact of the NLR on poor prognosis in patients with AIS treated with reperfusion therapy. Second, all analyzed studies were reported in English, which could have caused publication bias and influenced the pooled results. Hence, we used a “trim-and-fill” approach to reduce its influence on the effect size. Third, most of the studies were conducted in Asia, which may result in a risk of selection bias in the patient population. However, subgroup analyses found no effect of the study region on the research findings. Fourth, the statistically significant heterogeneity among the included studies may have affected the reliability of the meta-analysis, and thus, the conclusion should be more conservative. In the stratified subgroup analysis, none of the included factors was confirmed to be a contributing factor. Meanwhile, the results of sensitivity analyses showed that no single study affected the estimated significance of pooled ORs or SMDs. Discrepancies in various adjustments and inadequate consideration of potentially confounding factors may also partially explain the heterogeneity. Fifth, owing to the statistical characteristics of SMD, it was not possible to adjust for confounding factors (e.g., baseline NIHSS severity, hypertension, and age). Therefore, we attempted to include adjusted ORs for analysis in our study as much as possible. Sixth, the adjusted risk factors for each study used to calculate ORs were different in the included studies. However, almost all studies included key factors such as age, sex, and NIHSS scores. Seventh, statistical methods were used to calculate the approximation of the mean and SD from the median and IQR. These methods have been proven stable and reliable in previous studies. Eighth, a few studies were unable or did not seek to exclude patients with existing infections, which may affect the accuracy of NLR application. Nevertheless, there was no substantial difference in the effect size according to the subgroup analyses regardless of whether the infection was excluded. Ninth, NLR data were limited to two different time points in this analysis: admission/pre-treatment and post-treatment (time point close to 24 h). However, NLR dynamically changes during the progression of AIS. Thus, future analyses with more time points might further explore the relationship between the dynamic profile of the NLR and prognosis. Therefore, our findings should be interpreted with caution because of the above limitations.

## Conclusion

In summary, our findings show that both admission and post-treatment NLR can be used as cost-effective and easily available biomarkers to predict PFO at 3 months, sICH, and 3-month mortality in patients with AIS after reperfusion therapy. The predictive power of post-treatment NLR is better than that of admission. NLR as a stand-alone test or part of a risk prediction model may help clinicians easily and quickly identify patients after reperfusion therapy who have a poor prognosis and require more intensive monitoring during treatment. However, the prognostic value of the dynamic NLR is under investigation owing to the heterogeneity of the studies. Further studies are warranted to confirm the utility of the dynamic NLR in predicting the outcomes of patients with AIS treated with reperfusion therapy.

## Data availability statement

The original contributions presented in the study are included in the article/Supplementary material, further inquiries can be directed to the corresponding author.

## Author contributions

BW and FL: literature search, data extraction, statistical analysis, and drafting of the manuscript. GS and SW: study design, quality evaluation, and comments on important intellectual content. All authors have reviewed the manuscript. All authors contributed to the article and approved the submitted version.
